# Author Correction: Deep learning versus human graders for classifying diabetic retinopathy severity in a nationwide screening program

**DOI:** 10.1038/s41746-019-0146-5

**Published:** 2019-07-23

**Authors:** Paisan Ruamviboonsuk, Jonathan Krause, Peranut Chotcomwongse, Rory Sayres, Rajiv Raman, Kasumi Widner, Bilson J. L. Campana, Sonia Phene, Kornwipa Hemarat, Mongkol Tadarati, Sukhum Silpa-Archa, Jirawut Limwattanayingyong, Chetan Rao, Oscar Kuruvilla, Jesse Jung, Jeffrey Tan, Surapong Orprayoon, Chawawat Kangwanwongpaisan, Ramase Sukumalpaiboon, Chainarong Luengchaichawang, Jitumporn Fuangkaew, Pipat Kongsap, Lamyong Chualinpha, Sarawuth Saree, Srirut Kawinpanitan, Korntip Mitvongsa, Siriporn Lawanasakol, Chaiyasit Thepchatri, Lalita Wongpichedchai, Greg S. Corrado, Lily Peng, Dale R. Webster

**Affiliations:** 1 Department of Ophthalmology, Rajavithi Hospital, Bangkok, Thailand; 2 Google AI, Google, Mountain View, CA USA; 3 Shri Bhagwan Mahavir Vitreoretinal Services, Sankara Nethralaya, Chennai, Tamil Nadu India; 4 Department of Ophthalmology, Vajira Hospital, Bangkok, Thailand; 5 Eye and Laser Center, Charlotte, NC USA; 6 East Bay Retina Consultants, Oakland, CA; Department of Ophthalmology, University of California San Francisco, San Francisco, CA USA; 7 Retina-Vitreous Associates Medical Group, Department of Ophthalmology, University of Southern California, Los Angeles, CA USA; 8 Department of Ophthalmology, Lamphun Hospital, Lamphun, Thailand; 9 Department of Ophthalmology, Somdejphrajaotaksin Maharaj Hospital, Tak, Thailand; 10 Department of Ophthalmology, Sawanpracharak Hospital, Nakhon Sawan, Thailand; 11 Department of Ophthalmology, Nakhon Nayok Hospital, Nakhon Nayok, Thailand; 12 Department of Ophthalmology, Photharam Hospital, Ratchaburi, Thailand; 13 Department of Ophthalmology, Prapokklao Hospital, Chanthaburi, Thailand; 14 Department of Ophthalmology, Mahasarakham Hospital, Mahasarakham, Thailand; 15 Department of Ophthalmology, Nongbualamphu Hospital, Nongbualamphu, Thailand; 16 Department of Ophthalmology, Pakchongnana Hospital, Nakhon Ratchasima, Thailand; 17 Department of Ophthalmology, Mukdahan Hospital, Mukdahan, Thailand; 18 Department of Ophthalmology, Suratthani Hospital, Suratthani, Thailand; 19 Department of Ophthalmology, Sungaikolok Hospital, Narathiwat, Thailand; 20 Bangkok Metropolitan Administration Public Health Center 7, Bangkok, Thailand

**Keywords:** Diabetes complications, Developing world

**Correction to:** *npj Digital Medicine* 10.1038/s41746-019-0099-8, Published online 10 April 2019

The original version of the published Article contained an error in the spelling of the first Author’s name. “Paisan Raumviboonsuk” has been changed to “Paisan Ruamviboonsuk”. This has been corrected in the HTML and PDF version of the Article.

