# Supplementary material for: Deep learning versus human graders for classifying diabetic retinopathy severity in a nationwide screening program
Source: NPJ Digit Med. 2019 Apr 10;2:25. doi: 10.1038/s41746-019-0099-8 (PMC6550283; doi:10.1038/s41746-019-0099-8)
Supplement: Supplementary file 1 — Supplemental Material [file 41746_2019_99_MOESM1_ESM.pdf]

Deep Learning Versus Human Graders for Classifying Diabetic Retinopathy Severity in a Nationwide Screening Program -- Supplement

		Algorithm	
		DR Gradable	DR Ungradable
Regional Grader	DR Gradable	25,348	1,765
	DR Ungradable	1,239	1,591

		Algorithm	
		DME Gradable	DME Ungradable
Regional Grader	DME Gradable	24,332	2,467
	DME Ungradable	1,036	2,108

Supplementary Table 1. Agreement between regional graders and the algorithm DR and DME image gradability.

		Regional Grader	
		DR Gradable	DR Ungradable
Adjudication	DR Gradable	56	155
	DR Ungradable	547	224

		Algorithm	
		DR Gradable	DR Ungradable
Adjudication	DR Gradable	155	56
	DR Ungradable	224	547

Supplementary Table 2. **Agreement between adjudication and regional grader or algorithm for DR gradability.** Only images where the regional grader and the algorithm disagreed on DR gradability is included in this analysis.

		Regional Grader	
		DME Gradable	DME Ungradable
Adjudication	DME Gradable	493	277
	DME Ungradable	196	28

		Algorithm	
		DME Gradable	DME Ungradable
Adjudication	DME Gradable	277	493
	DME Ungradable	28	196

Supplementary Table 3. Agreement between adjudication and regional grader or algorithm for DME gradability. Only images where the regional grader and the algorithm disagreed on DR gradability is included in this analysis.

	Moderate or Worse DR						Severe or Worse DR						Proliferative DR						DME					
		Sensitivity		Specificity		AUC		Sensitivity		Specificity		AUC		Sensitivity		Specificity		AUC		Sensitivity		Specificity		AUC
Regional Grader Expertise	n	Regional Grader	Algorithm	Regional Grader	Algorithm	Algorithm	n	Regional Grader	Algorithm	Regional Grader	Algorithm	Algorithm	n	Regional Grader	Algorithm	Regional Grader	Algorithm	Algorithm	n	Regional Grader	Algorithm	Regional Grader	Algorithm	Algorithm
(All regions)	3,069	73.48 [72, 75.03]	96.87 [96, 97.46]	98.09 [98, 98.26]	95.63 [95, 95.89]	98.78 [98.67, 98.91]	599	60.60 [57, 64.54]	93.32 [91, 95.19]	99.70 [100, 99.77]	97.59 [97, 97.78]	99.16 [99.01, 99.30]	394	61.17 [56, 66.01]	72.34 [68, 76.70]	99.78 [100, 99.83]	99.85 [100, 99.89]	99.38 [99.15, 99.60]	1,843	62.07 [60, 64.29]	95.23 [94, 96.15]	99.24 [99, 99.35]	98.21 [98, 98.38]	99.39 [99.32, 99.42]
Nurse or Technician	288	44.10 [38, 50.04]	99.31 [98, 99.92]	99.44 [99, 99.69]	94.49 [94, 95.36]	99.16 [98.89, 99.34]	62	69.35 [56, 80.44]	95.16 [87, 98.99]	99.30 [99, 99.58]	98.08 [97, 98.57]	99.53 [99.27, 99.62]	26	57.69 [37, 76.65]	69.23 [48, 85.67]	99.24 [99, 99.53]	99.85 [100, 99.96]	99.42 [99.11, 99.60]	194	45.36 [38, 52.65]	91.24 [86, 94.81]	98.93 [98, 99.30]	98.93 [98, 99.30]	99.38 [99.24, 99.60]
Nurse or Technician	113	73.45 [64, 81.32]	96.46 [91, 99.03]	96.53 [95, 97.43]	98.70 [98, 99.23]	99.51 [99.34, 99.67]	22	72.73 [50, 89.27]	81.82 [60, 94.81]	99.46 [99, 99.77]	98.71 [98, 99.22]	99.22 [98.74, 99.75]	17	82.35 [57, 96.20]	70.59 [44, 89.69]	99.66 [99, 99.89]	99.93 [100, 100.00]	99.44 [99.04, 99.94]	74	58.11 [46, 69.49]	98.65 [93, 99.97]	98.31 [97, 98.92]	99.19 [99, 99.60]	99.89 [99.79, 99.96]
Nurse or Technician	117	91.45 [85, 95.83]	99.15 [95, 99.98]	97.29 [96, 98.32]	94.59 [93, 96.07]	99.46 [99.28, 99.54]	23	52.17 [31, 73.18]	100.00 [85, 100.00]	99.77 [99, 99.97]	96.67 [95, 97.76]	99.65 [99.45, 99.90]	11	27.27 [6, 60.97]	90.91 [59, 99.77]	99.89 [99, 100.00]	99.89 [99, 100.00]	99.51 [98.50, 100.00]	60	68.33 [55, 79.74]	96.67 [88, 99.59]	98.82 [98, 99.46]	98.30 [97, 99.09]	99.64 [99.20, 99.80]
Nurse or Technician	339	89.38 [86, 92.45]	94.10 [91, 96.36]	93.95 [92, 95.32]	95.59 [94, 96.75]	98.45 [98.10, 98.88]	65	56.92 [44, 69.15]	96.92 [89, 99.63]	99.77 [99, 99.95]	94.53 [93, 95.69]	98.87 [98.13, 99.12]	49	57.14 [42, 71.18]	73.47 [59, 85.05]	99.77 [99, 99.95]	99.85 [99, 99.98]	99.66 [99.34, 99.71]	243	58.85 [52, 65.10]	98.35 [96, 99.55]	99.61 [99, 99.89]	96.21 [95, 97.29]	99.38 [99.06, 99.53]
Nurse or Technician	218	66.51 [60, 72.74]	92.66 [88, 95.75]	99.45 [99, 99.67]	97.10 [96, 97.64]	97.74 [96.92, 98.36]	27	77.78 [58, 91.38]	96.30 [81, 99.91]	99.94 [100, 99.99]	99.28 [99, 99.53]	99.62 [99.18, 99.94]	14	78.57 [49, 95.34]	92.86 [66, 99.82]	99.94 [100, 99.99]	99.97 [100, 100.00]	99.95 [99.85, 100.00]	89	65.17 [54, 74.96]	97.75 [92, 99.73]	99.73 [99, 99.88]	98.67 [98, 99.03]	99.71 [99.65, 99.82]
Nurse or Technician	347	63.40 [58, 68.48]	97.12 [95, 98.61]	98.99 [98, 99.41]	94.13 [93, 95.21]	98.60 [98.21, 98.95]	94	32.98 [24, 43.44]	92.55 [85, 96.95]	99.85 [100, 99.97]	95.46 [94, 96.35]	98.23 [97.87, 98.94]	65	26.15 [16, 38.54]	60.00 [47, 71.96]	100.00 [100, 100.00]	99.75 [99, 99.92]	98.53 [97.77, 99.58]	235	56.17 [50, 62.61]	100.00 [98, 100.00]	99.75 [99, 99.93]	97.74 [97, 98.41]	99.72 [99.59, 99.76]
Ophthalmologist	125	54.40 [45, 63.33]	98.40 [94, 99.81]	99.54 [99, 99.79]	94.96 [94, 95.89]	98.86 [98.30, 99.16]	13	30.77 [9, 61.43]	84.62 [55, 98.08]	99.90 [100, 99.99]	99.13 [99, 99.49]	99.49 [98.99, 99.90]	8	50.00 [16, 84.30]	50.00 [16, 84.30]	99.90 [100, 99.99]	100.00 [100, 100.00]	98.96 [97.89, 99.95]	78	50.00 [38, 61.54]	96.15 [89, 99.20]	99.85 [100, 99.97]	99.24 [99, 99.58]	99.81 [99.65, 99.89]
Ophthalmologist	650	95.54 [94, 96.99]	98.31 [97, 99.15]	95.84 [95, 96.53]	96.01 [95, 96.68]	98.96 [98.82, 99.20]	61	65.57 [52, 77.27]	96.72 [89, 99.60]	99.94 [100, 99.99]	97.10 [97, 97.62]	99.31 [98.87, 99.48]	41	78.05 [62, 89.44]	73.17 [57, 85.78]	99.95 [100, 99.99]	99.86 [100, 99.96]	99.74 [99.58, 99.85]	349	79.66 [75, 83.76]	85.96 [82, 89.43]	99.26 [99, 99.52]	98.36 [98, 98.77]	98.62 [98.34, 99.05]
Ophthalmologist	258	40.70 [35, 46.96]	97.29 [94, 98.90]	99.10 [99, 99.47]	93.78 [93, 94.80]	98.78 [98.56, 99.17]	55	78.18 [65, 88.19]	90.91 [80, 96.98]	99.28 [99, 99.59]	97.47 [97, 98.08]	99.02 [98.37, 99.59]	38	73.68 [57, 86.60]	65.79 [49, 80.37]	99.55 [99, 99.78]	99.69 [99, 99.87]	99.44 [99.01, 99.93]	179	58.10 [51, 65.42]	98.88 [96, 99.86]	97.32 [97, 97.99]	97.47 [97, 98.12]	99.59 [99.48, 99.73]
Ophthalmologist	226	83.63 [78, 88.20]	98.67 [96, 99.73]	97.33 [95, 98.57]	90.35 [87, 92.82]	98.20 [97.19, 98.63]	56	60.71 [47, 73.50]	98.21 [90, 99.95]	99.70 [99, 99.96]	91.48 [89, 93.50]	98.83 [98.35, 99.35]	33	78.79 [61, 91.02]	84.85 [68, 94.89]	99.85 [99, 100.00]	99.41 [99, 99.84]	99.26 [98.54, 99.76]	132	80.30 [72, 86.71]	95.45 [90, 98.31]	99.11 [98, 99.71]	94.49 [92, 96.23]	98.05 [97.44, 98.83]
Ophthalmologist	100	67.00 [57, 76.08]	97.00 [91, 99.38]	98.05 [97, 98.72]	96.32 [95, 97.27]	97.96 [96.51, 99.13]	26	65.38 [44, 82.79]	92.31 [75, 99.05]	99.36 [99, 99.71]	98.51 [98, 99.07]	97.81 [94.44, 99.88]	15	46.67 [21, 73.41]	80.00 [52, 95.67]	99.51 [99, 99.80]	99.86 [99, 99.98]	97.48 [93.97, 99.97]	58	63.79 [50, 76.01]	98.28 [91, 99.96]	99.56 [99, 99.86]	97.43 [96, 98.27]	99.31 [99.24, 99.61]
Ophthalmologist	150	90.00 [84, 94.29]	89.33 [83, 93.78]	95.15 [94, 96.35]	98.35 [97, 99.02]	98.78 [98.16, 98.87]	58	70.69 [57, 81.91]	84.48 [73, 92.65]	99.66 [99, 99.91]	97.97 [97, 98.70]	98.90 [98.65, 99.44]	51	74.51 [60, 85.67]	68.63 [54, 80.89]	99.83 [99, 99.98]	99.75 [99, 99.95]	99.37 [99.24, 99.69]	81	53.09 [42, 64.27]	98.77 [93, 99.97]	99.82 [99, 99.98]	97.05 [96, 97.97]	99.60 [99.47, 99.76]
Ophthalmologist	138	61.59 [53, 69.74]	99.28 [96, 99.98]	100.00 [100, 100.00]	95.43 [94, 96.38]	99.66 [99.52, 99.76]	37	64.86 [47, 79.79]	94.59 [82, 99.34]	99.94 [100, 100.00]	98.32 [98, 98.86]	99.73 [99.47, 99.85]	26	69.23 [48, 85.67]	88.46 [70, 97.55]	100.00 [100, 100.00]	99.83 [100, 99.97]	99.84 [99.67, 99.95]	71	45.07 [33, 57.34]	100.00 [95, 100.00]	99.89 [100, 99.99]	98.86 [98, 99.30]	99.79 [99.70, 99.85]

Supplementary Table 4. Performance of grader and algorithm by region for DR and DME for all gradable images. The order of regions reported is randomized to partially anonymize regional graders' metrics. n=Number of positive cases for each condition in each region.

Regional Grader Expertise	Regional Grader Kappa	Algorithm Kappa
(All regions)	77.35 [71.16, 72.39]	84.44 [71.63, 73.80]
Nurse or Technician	64.16 [50.88, 54.74]	82.42 [66.23, 72.72]
Nurse or Technician	74.18 [57.49, 67.34]	86.63 [75.68, 86.42]
Nurse or Technician	80.35 [76.18, 85.10]	86.17 [66.05, 72.56]
Nurse or Technician	81.29 [73.62, 79.13]	87.09 [70.04, 76.85]
Nurse or Technician	80.83 [66.30, 75.59]	82.88 [68.60, 74.36]
Nurse or Technician	65.82 [57.70, 64.94]	84.47 [65.55, 69.48]
Ophthalmologist	62.41 [59.74, 70.98]	73.66 [58.94, 67.81]
Ophthalmologist	87.45 [83.26, 85.95]	85.77 [77.74, 80.59]
Ophthalmologist	68.60 [47.12, 56.06]	78.52 [63.58, 70.70]
Ophthalmologist	86.23 [71.54, 83.18]	85.58 [66.94, 74.23]
Ophthalmologist	83.81 [68.52, 75.12]	86.02 [74.09, 83.95]
Ophthalmologist	80.86 [70.10, 78.20]	84.95 [63.03, 69.35]
Ophthalmologist	67.08 [56.16, 69.57]	82.26 [63.75, 74.08]

Supplementary Table S5. Performance of the grader compared to the algorithm by region for DR. Agreement is measured in terms of quadratic weighted kappa and unweighted kappa for the 4 categories of DR (No/Mild, Moderate, Severe, Proliferative)

Camera Make	Camera Models
3nethra	Classic
Canon	CR-2
Kowa	VX-10, VX-20, Nonmyd 7, Nonmyd WD, Nonmyd a-D III 8300
Nidek	AFC-210, AFC-230, AFC-300
Topcon	TRC NW-8
Zeiss	Visucam 200

Supplementary Table 6. Camera make and models used in the study. 31 total cameras (1-6 per region) were used to acquire the fundus images.

DR images flowchart

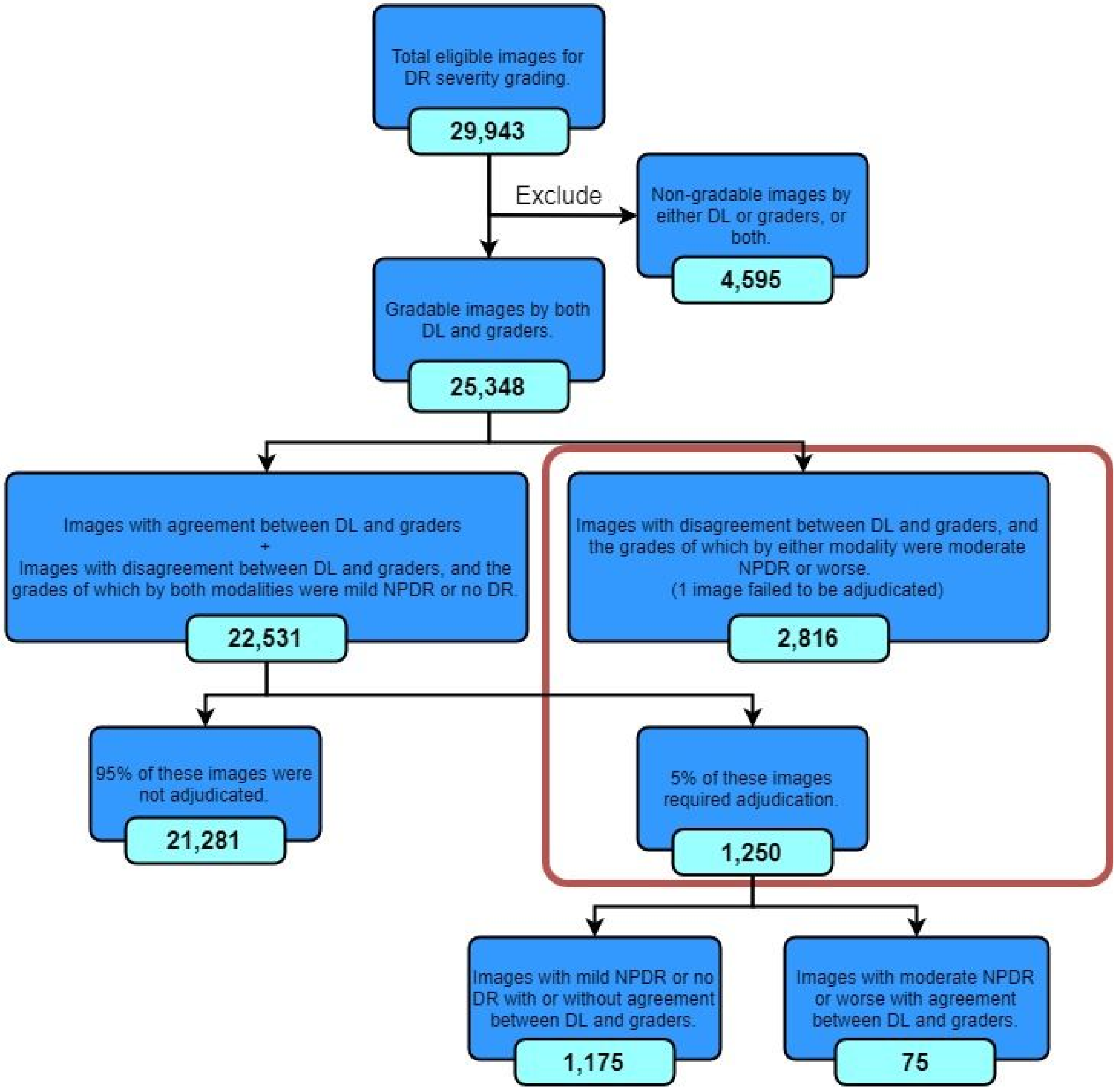

Supplementary Figure 1. Flowchart of images eligible for adjudication for DR severity levels by retinal specialists according to study protocols. The criteria and number of images for the adjudication were within the red rounded rectangle.

DME images flowchart

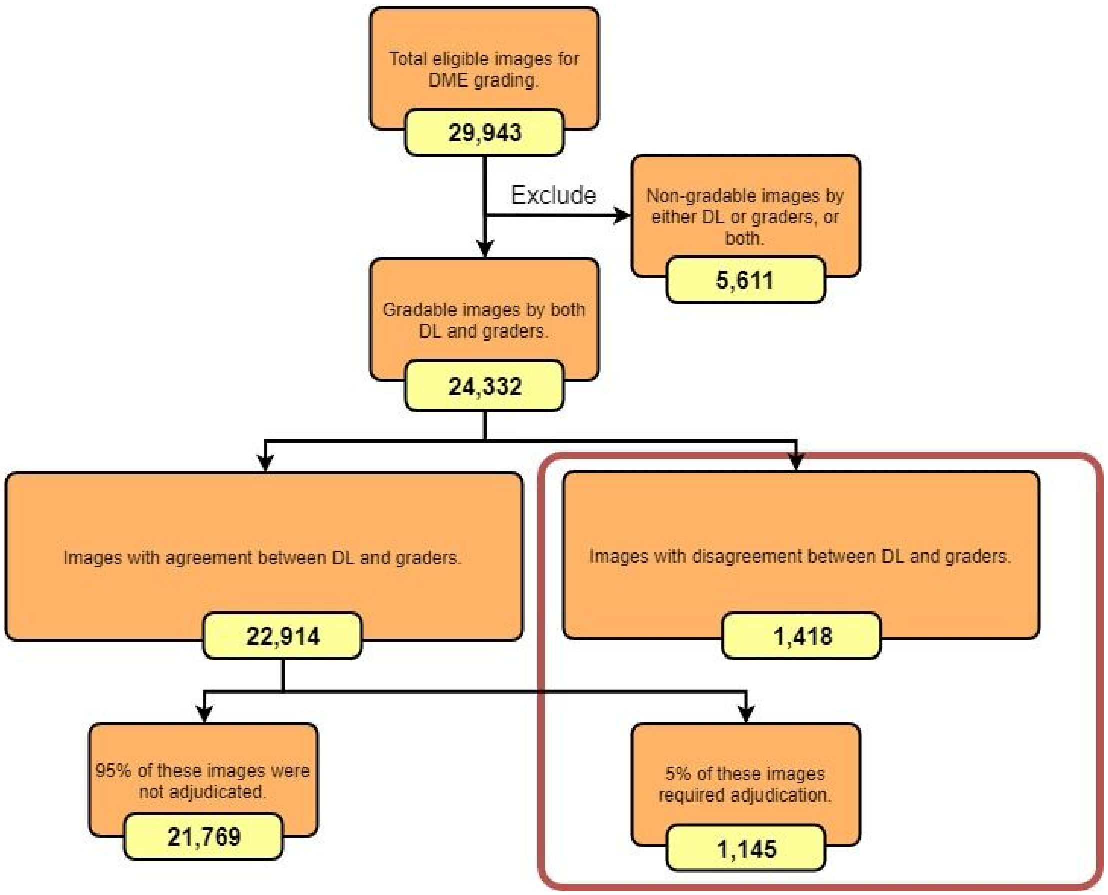

Supplementary Figure 2. Flowchart of images eligible for adjudication for DR severity levels by retinal specialists according to study protocols. The criteria and number of images for the adjudication were within the red rounded rectangle.

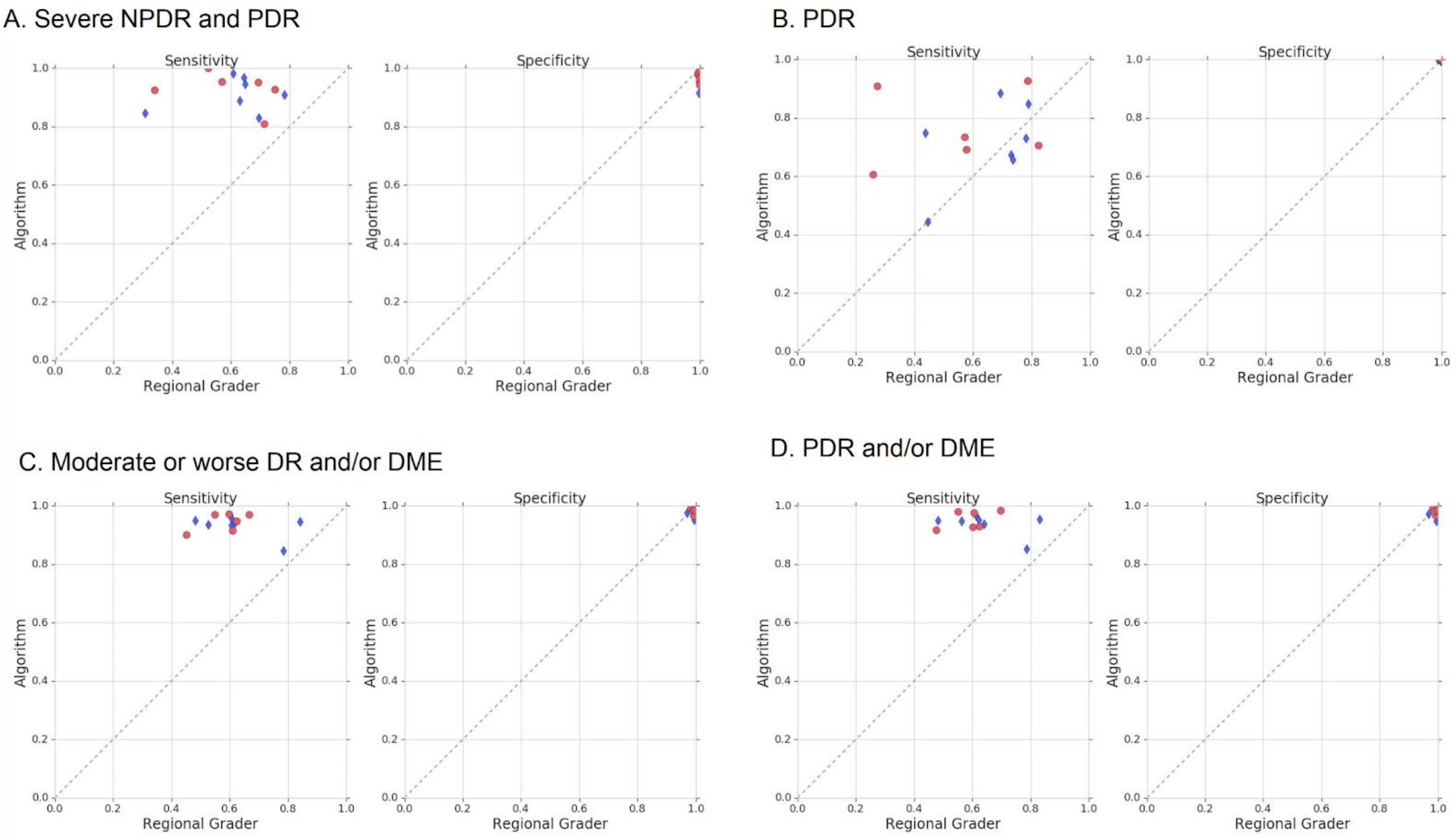

Supplementary Figure 3. Performance of algorithm and regional graders for varying and combination of DR and DME. Grader performances are represented as blue diamonds (ophthalmologists) and red dots (nurse or technician) for (A) Severe NPDR and PDR, (B) PDR, (C) Moderate or worse DR and/or DME, (D) PDR and/or DME. Analysis is performed on all gradable images. The rest of the combinations are in Figure 2 of the main manuscript.

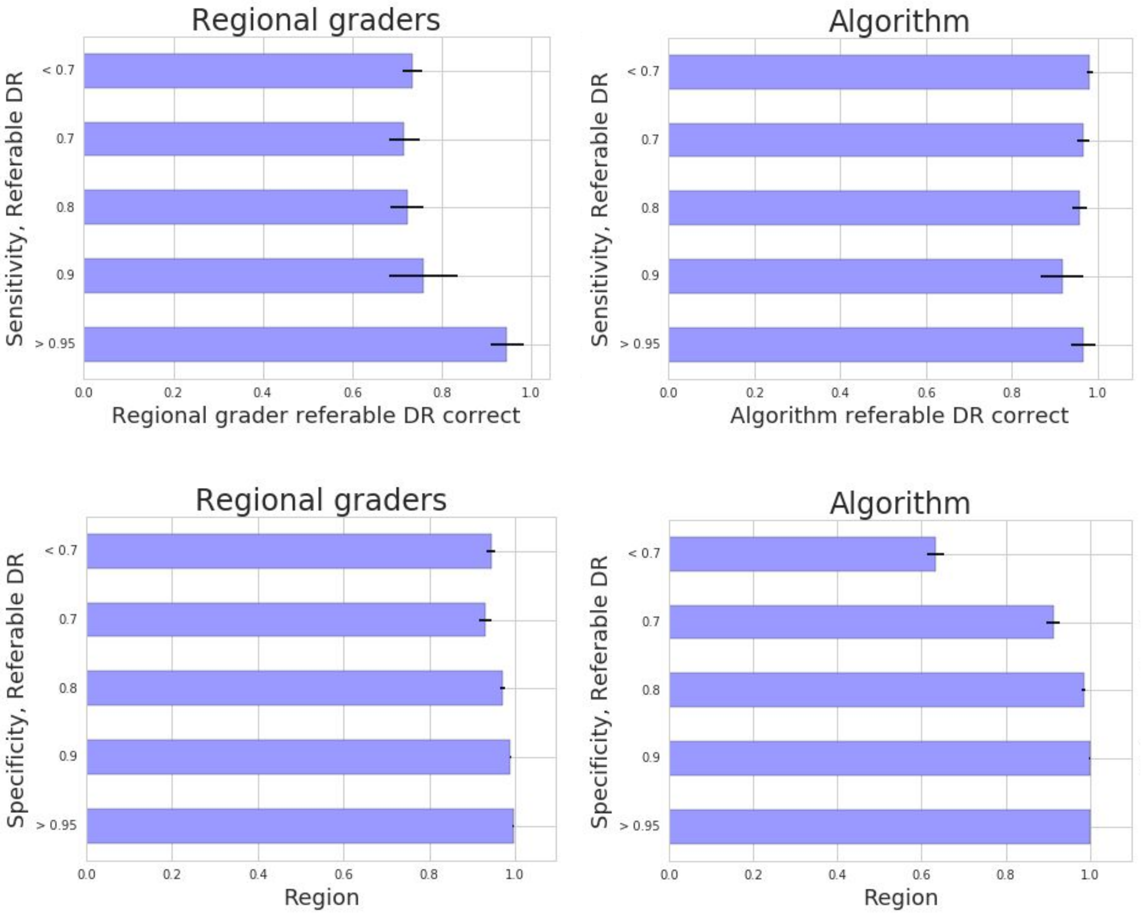

Supplementary Figure 4. Comparison of regional grader and algorithm sensitivity and specificity based on algorithm maximum confidence score. The algorithm is more sensitive than regional graders at all levels of confidence but less specific for low-confidence images (where the algorithm confidence scores <0.7).

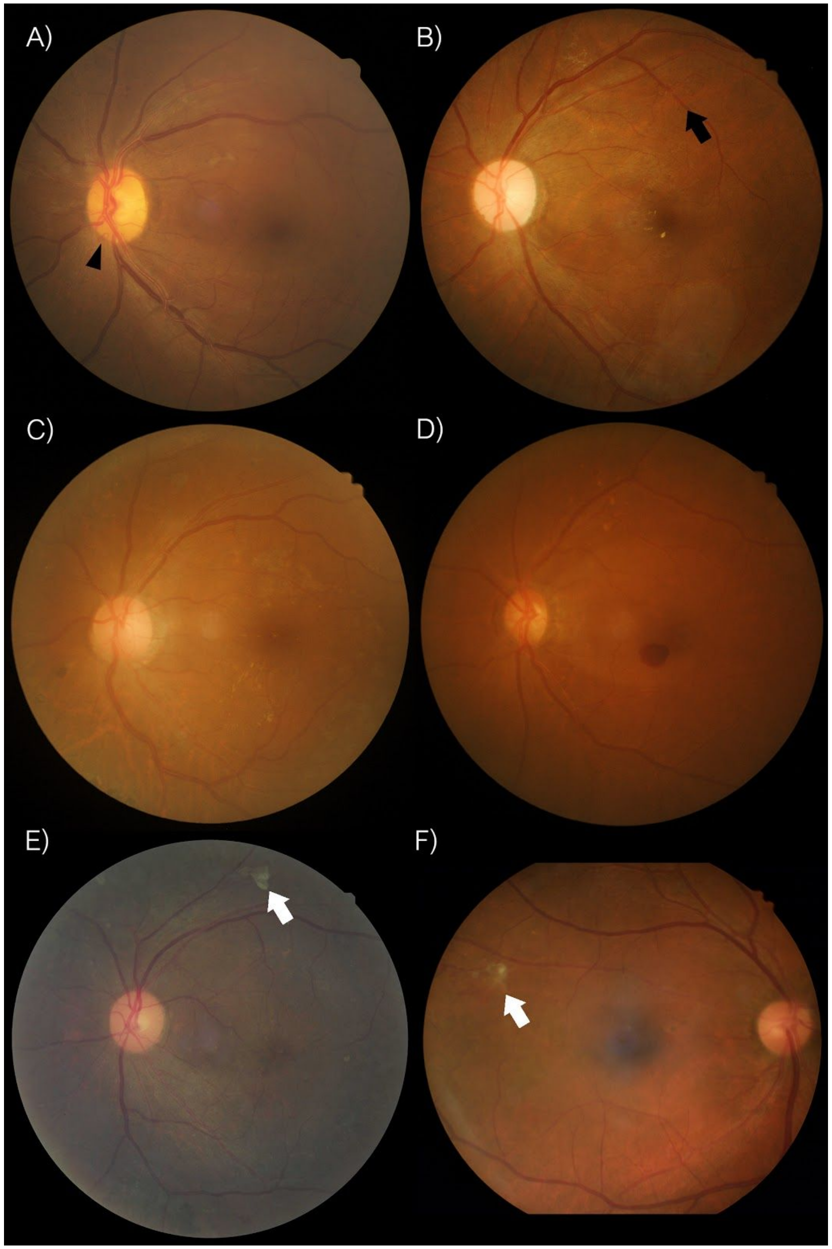

Supplementary Figure 5. **Sample of cases missed by the algorithm and regional grader**. Figures A) and B) show obscure neovascularization at disc and elsewhere respectively. Figure C) contains panretinal photocoagulation scars at the edge of the image. Figure D) contains preretinal hemorrhage. Figures E) and F) contain inactive fibrous tissue.
